# Serum Glycated Albumin to Guide the Diagnosis of Diabetes Mellitus

**DOI:** 10.1371/journal.pone.0146780

**Published:** 2016-01-14

**Authors:** Wan-Chen Wu, Wen-Ya Ma, Jung-Nan Wei, Tse-Ya Yu, Mao-Shin Lin, Shyang-Rong Shih, Cyue-Huei Hua, Ying-Jhu Liao, Lee-Ming Chuang, Hung-Yuan Li

**Affiliations:** 1 Division of Endocrinology, Department of Internal Medicine, National Taiwan University Hospital, Taipei, Taiwan; 2 Division of Endocrinology, Department of Internal Medicine, Cardinal Tien Hospital, New Taipei City, Taiwan; 3 Chia Nan University of Pharmacy and Science, Tainan, Taiwan; 4 Department of Health Management Center, Far Eastern Memorial Hospital, New Taipei City, Taiwan; 5 Division of Cardiology, Department of Internal Medicine, National Taiwan University Hospital, Taipei, Taiwan; 6 Division of Clinical Pathology, National Taiwan University Hospital Yun-Lin Branch, Yun-Lin, Taiwan; GDC, GERMANY

## Abstract

In the diagnosis of diabetes mellitus, hemoglobin A1c (HbA1c) is sometimes measured to determine the need of an oral glucose tolerance test (OGTT). However, HbA1c does not accurately reflect glycemic status in certain conditions. This study was performed to test the possibility that measurement of serum glycated albumin (GA) better assesses the need for OGTT. From 2006 to 2012, 1559 subjects not known to have diabetes or to use anti-diabetic medications were enrolled. Serum GA was measured, and a 75-g OGTT was then performed to diagnose diabetes. Serum GA correlated significantly to age (r = 0.27, p<0.001), serum albumin (r = –0.1179, age-adjusted p = 0.001), body mass index (r = -0.24, age-adjusted p<0.001), waist circumference (r = -0.16, age-adjusted p<0.001), and plasma GA (r = 0.999, p<0.001), but was unaffected by diet (p = 0.8). Using serum GA at 15% for diagnosis of diabetes, the sensitivity, specificity, and area under the receiver-operating characteristic curve were 74%, 85%, and 0.86, respectively. Applying a fasting plasma glucose (FPG) value of < 100 mg/dL to exclude diabetes and of ≥ 126 mg/dL to diagnose diabetes, 14.4% of the study population require an OGTT (OGTT%) with a sensitivity of 78.8% and a specificity of 100%. When serum GA value of 14% and 17% were used to exclude and diagnose diabetes, respectively, the sensitivity improved to 83.3%, with a slightly decrease in specificity (98.2%), but a significant increase in OGTT% (35%). Using combined FPG and serum GA cutoff values (FPG < 100 mg/dL plus serum GA < 15% to exclude diabetes and FPG ≥ 126 mg/dL or serum GA ≥ 17% to diagnose diabetes), the OGTT% was reduced to 22.5% and the sensitivity increased to 85.6% with no change in specificity (98.2%). In the diagnosis of diabetes, serum GA measurements can be used to determine the need of an OGTT.

## Introduction

In accordance with the recommendations of the American Diabetes Association (ADA), International Diabetes Federation, and World Health Organization [1–4], diagnosis of diabetes mellitus should be based on either an oral glucose tolerance test (OGTT) or hemoglobin A1c (HbA1c) findings. However, the OGTT is time-consuming and requires two blood samplings. In the diagnosis of diabetes, therefore, HbA1c values are often used to access the need for an OGTT [5–9]. For example, subjects with fasting plasma glucose 100–125 mg/dl or HbA1c 6.1–6.4% were recommended to receive OGTT, according to the clinical practice guidelines by Diabetes Association of the R.O.C. in 2015 [9], which is modified based on our previous report [5]. Nonetheless, measurement of HbA1c may not accurately reflect blood glucose concentrations in certain conditions. For example, lower HbA1c values are found in conditions associated with a decrease erythrocyte lifespan, such as recent transfusion and increased erythropoiesis secondary to hemolysis or blood loss [10–12]. In patients with chronic kidney disease (CKD), erythrocyte lifespan is decreased by anemia, recent transfusions, and other alterations which result in reduced HbA1c values. In these patients, carbamylated hemoglobin is recognized to interfere with HbA1c measurements [11]. HbA1c values are reported to be higher in conditions, such as asplenia, wherein erythrocyte lifespan increases [12]. Iron deficiency anemia is also associated with higher HbA1c value due to the enhancement of hemoglobin glycation by increased plasma malondialdehyde [13, 14]. Additionally, genetic variants of hemoglobin, such as HbS trait and HbC trait, and elevated fetal hemoglobin (HbF) concentration can affect the accuracy of HbA1c measurements. Based on the prevalence of anemia and chronic kidney disease, up to one fourth of the population is estimated to have a condition wherein HbA1c values do not accurately reflect blood glucose concentrations [15–17]. Therefore, an alternative procedure to determine the need for an OGTT in the diagnosis of diabetes is needed.

Glycated albumin (GA), a ketoamine formed by non-enzymatic glycation of serum albumin, reflects short-term (2–3 weeks) mean glycemic levels [18, 19]. GA values are not affected by changes in erythrocyte lifespan [20], and measurement of GA is not influenced by anemia or other conditions which invalidate HbA1c measurements in the diagnosis of diabetes [18]. Furthermore, GA is reported to serve as a better indicator of glycemic control than HbA1c in diabetic patients on dialysis [21–23]. Findings from the DCCT/EDIC and other studies [24–28] revealed the association of increased GA values with the presence of diabetic retinopathy, nephropathy, and cardiovascular complications; these findings also support the use of GA measurements in the diagnosis of diabetes. The present study was undertaken to ascertain whether measurement of GA can be employed to determine the need for an OGTT in the diagnosis of diabetes.

## Methods

### Study subjects

A community-based cohort study, termed the Taiwan Lifestyle Study, was conducted. From 2006 to 2012, subjects older than 18 years were invited to participate in the study [5, 29, 30]. All participates completed a questionnaire, underwent a physical examination, and received blood tests. Subjects with diabetes or who were receiving anti-diabetic drugs were excluded. Written informed consent was obtained from each participant. This study was reviewed and approved by the Institutional Review Board of the National Taiwan University Hospital.

Body height (to the nearest 0.5 cm) and body weight (to the nearest 0.1 kg) were measured by attending nurses. Body mass index (BMI) was calculated by dividing body weight (kg) by the square of body height (m). Waist circumference (WC) was measured in the horizontal plane midway between lowest rib and the iliac crest [31].

### The oral glucose tolerance test

A standard 75-g OGTT was performed after a fast at least 8 hours. Blood samples were collected and stored at -80°C for serological assay. Normal glucose tolerance (fasting plasma glucose [FPG] < 5.56 mmol/L and 2-h plasma glucose [PG] < 7.8 mmol/L), impaired fasting glucose (IFG) (FPG = 5.56–6.9 mmol/L), impaired glucose tolerance (2-h PG = 7.8–11.0 mmol/L), and diabetes mellitus (FPG ≥ 7 mmol/L and/or 2-h PG ≥ 11.1 mmol/L) were defined according to the ADA diagnostic criteria [32]. GA was measured using the LUCICA GA-L kit (Asahi Kasei Pharma, Tokyo, Japan) and the Beckman Coulter AU2700 Chemistry Analyzer (Beckman Coulter Systems Co., Nyon, Switzerland). GA in paired serum and plasma samples from twelve subjects were measured to determine whether the method of sample collection affected GA measurements. Plasma glucose and HbA1c were measured using automatic analyzers (Toshiba TBA 120FR, Toshiba Medical Systems Co., Tokyo, Japan and HLC-723 G7 HPLC systems, Tosoh Corporation, Tokyo, Japan, respectively). The HbA1c assay was certified by the National Glycohemoglobin Standardization Program [33] and standardized to the DCCT reference assay.

### Statistical analyses

Findings are presented as means ± standard deviations (SDs) for continuous variables and as percentages for categorical variables. The differences between the GA < 14% and the GA ≥ 14% groups in Table 1 were determined using the Student's t test or chi-square test. The performance of serum GA in the diagnosis of diabetes by the OGTT was analyzed by the receiver operating characteristic (ROC) curve. The optimal cutoff for serum GA was derived from the ROC curve with the shortest distance to sensitivity = 1 and 1–specificity = 0 and with the Youden index (Y = sensitivity + specificity– 1). The performance of different criteria (from GA of 10.0% to 23.0%, with a 0.1% intervals) to diagnose diabetes was calculated and included the proportion of subjects receiving an OGTT (OGTT%), sensitivity, specificity, positive predictive value (PPV), negative predictive value (NPV), false negative rates (FNR), and false positive rates (FPR). Optimal cutoffs with minimal OGTT% were chosen among cutoffs with sensitivity > 80% and specificity > 95%. The difference in serum GA values between men and women was determined by the Student's t test. Differences between serum and plasma GA values and between fasting and postprandial GA values were determined by the paired t test. The relationships of serum GA to age, serum albumin, body mass index (BMI), waist circumference (WC), and plasma GA were analyzed by Pearson's correlation coefficients and linear regression models. The relationships of serum GA to FPG, to 2-hr PG after the OGTT, and to HbA1c were analyzed by quadratic regression models. A two-tailed *p* value < 0.05 was considered to be statistically significant. The statistical analyses were performed with STATA/SE 11 for Windows (StataCorp LP, College Station, TX, USA).

**Table 1:** Clinical characteristics of the study participants by the mean of serum glycated albumin.

	All	Serum glycated albumin		
		< 14%	≥ 14%	*p*
*n*	1559	925	634	
Age (years)	50.4 ± 12.6	47.9 ± 12.3	54.0 ± 12.1	<0.001
Sex (male/female)	624/935	393/532	231/403	0.017
Family history of diabetes (N, %)	590	352 (38.1%)	238 (37.5%)	0.860
BMI (kg/m^2^)	24.3 ± 3.4	24.6 ± 3.4	23.8 ± 3.4	<0.001
WC (cm)	81.8 ± 10.3	82.7 ± 10.3	81.0 ± 10.2	0.002
FPG (mg/dL, mmol/L)	93 ± 19 (5.2 ± 1.1)	89 ± 8 (5.0 ± 0.5)	100 ± 27 (5.5 ± 1.5)	<0.001
2-h PG (mg/dL, mmol/L)	127 ± 58 (7.0 ± 3.2)	114 ± 35 (6.3 ± 1.9)	146 ± 76 (8.1 ± 4.2)	<0.001
HbA1c (%, mmol/mol)	5.8 ± 0.8 (39.7 ± 8.9)	5.6 ± 0.4 (37.6 ± 4.4)	6.1 ± 1.1 (42.8 ± 12.2)	<0.001
Serum glycated albumin (%)	14.0 ± 2.6	12.7 ± 0.9	15.8 ± 3.2	<0.001
Serum creatinine (mg/dL)	1.0 ± 0.2	1.0 ± 0.2	1.0 ± 0.2	0.51
By status of diabetes				<0.001
NGT (N, %)	1041 (66.8%)	693 (74.9%)	348 (35.6%)	
IFG or IGT (N, %)	386 (24.8%)	210 (25.1%)	176 (45.0%)	
Diabetes (N, %)	132 (8.5%)	14 (1.5%)	118 (18.6%)	

Means ± SDs were shown for continuous variables. BMI, body mass index; FPG, fasting plasma glucose; 2-h PG, plasma glucose 2 h after oral glucose tolerance test; HbA1c, hemoglobin A1c; WC, waist circumference. NGT, normal glucose tolerance; IFG, impaired fasting glucose; IGT, impaired glucose tolerance.

## Results

A total of 1559 participants were enrolled in the present study, including 624 (40%) men and 935 (60%) women. Mean ± SD for age, BMI, and WC were 50.4 ± 12.6 years, 24.3 ± 3.4 kg/m^2^, and 81.8 ± 10.3 cm, respectively. Serum GA distributed normally (mean 14%, SD 2.6%, 95% CI 11.4%–17.1%). Mean ± SD for FPG, 2-h PG, and HbA1c for these subjects were 5.2 ± 1.1 mmol/L, 7.0 ± 3.2 mmol/L, and 5.8 ± 0.8%, respectively. Clinical characteristics for the study group are summarized in Table 1. Subjects with serum GA measurement ≥ 14% were older and had higher FPG, 2-h PG, and HbA1c values as compared to those below 14%. Additionally, the male/female ratio was lower in the group with serum GA measurements ≥ 14%.

Factors potentially associated with the measurement of serum GA were examined. The relationship of serum GA to age, gender, serum albumin, BMI, and WC in subjects without diabetes is presented in S1 Fig. No difference in serum GA was observed as a function of gender (14.0 ± 2.6% for females and 13.9 ± 2.6% for males, p = 0.26). Serum GA was associated with age in these subjects (r = 0.27, p < 0.001). Ten—year interval increases in age were each associated with a 0.31% increase in serum GA. Serum GA was negatively associated with serum albumin in these subjects (r = -0.12, age-adjusted p = 0.001). For every 1 g/dl increase in serum albumin, serum GA was observed to decrease by of 0.32%. Serum GA was inversely correlated with anthropometric parameters, including BMI and WC, in subjects without diabetes (r = -0.24 with age-adjusted p < 0.001 and r = -0.16 with age-adjusted p < 0.001, respectively). Each 1 kg/m^2^ increase in BMI was associated with a 0.12% decrease in serum GA, and each 1 cm increase in WC was associated with a decrease in serum GA of 0.04%. The influence of sample collection method on GA measurements in non-diabetic subjects is presented in S2 Fig. Serum GA was closely correlated to plasma GA (r = 0.999, p < 0.001; serum GA = 0.98 x plasma GA + 0.29). Furthermore, serum and plasma GA values did not change in sample collection during a fast, postprandial, or after glucose challenge (mean serum GA of 17.9 ± 6.7% during a fast and mean serum GA of 18.0 ± 6.5% at 2 h after glucose challenge, p = 0.8; mean plasma GA of 15.8 ± 4.3% during a fasting and mean plasma GA of 15.8 ± 4.3% at 2 h post-prandial, p = 0.8).

Quadratic relationships between serum GA and FPG, 2-h PG, and HbA1c were observed for subjects with and without diabetes (S3 Fig). Table 2 shows the numeric relationships between serum GA and FPG, 2-h PG, and HbA1c. According to these quadratic relationship, serum GA of 14% corresponds to a HbA1c value of 5.7% whereas serum GA of 18% corresponds to a HbA1c value of 6.5%.

**Table 2:** The relationship between serum glycated albumin, hemoglobin A1c, fasting plasma glucose, and plasma glucose 2h after oral glucose tolerance test.

Serum GA (%)	Hemoglobin A1c (%, mmol/mol)	FPG (mg/dL, mmol/L)	OGTT 2h PG (mmol/L)
13	5.6 (38)	89 (5.0)	114 (6.3)
**14**	**5.7 (39)**	**92 (5.1)**	**125 (7.0)**
**15**	**5.9 (41)**	**96 (5.3)**	**137 (7.6)**
16	6.1 (43)	99 (5.5)	149 (8.3)
**17**	**6.3 (45)**	**104 (5.8)**	**162 (9.0)**
**18**	**6.5 (48)**	**108 (6.0)**	**175 (9.7)**
19	6.7 (50)	113 (6.3)	189 (10.5)
20	6.9 (52)	118 (6.6)	203 (11.3)
**20.5**	**7.0 (53)**	**121 (6.7)**	**210 (11.7)**
21	7.1 (54)	124 (6.9)	218 (12.1)
22	7.4 (57)	130 (7.2)	233 (12.9)
23	7.7 (61)	136 (7.5)	248 (13.8)
24	7.9 (63)	143 (7.9)	265 (14.7)
25	8.2 (66)	150 (8.3)	281 (15.6)
26	8.5 (69)	157 (8.7)	298 (16.6)
27	8.8 (73)	165 (9.1)	316 (17.5)
28	9.2 (77)	173 (9.6)	334 (18.5)
29	9.5 (80)	181 (10.1)	352 (19.6)
30	9.8 (84)	190 (10.6)	371 (20.6)

FPG, fasting plasma glucose; GA, glycated albumin; OGTT 2h PG, plasma glucose 2 hours after oral glucose tolerance tests.

The area under the ROC curve for serum GA in the diagnosis of diabetes by the OGTT was 0.86 (95% CI 0.82–0.90) whereas the area under the ROC curve for HbA1c in the diagnosis of diabetes by the OGTT was 0.90 (95% CI 0.86–0.94). The optimal cutoff value for serum GA was 15%; the sensitivity, specificity, PPV, and NPV for serum GA in the diagnosis of diabetes were 74%, 85%, 32%, and 97%, respectively.

**Fig 1:**
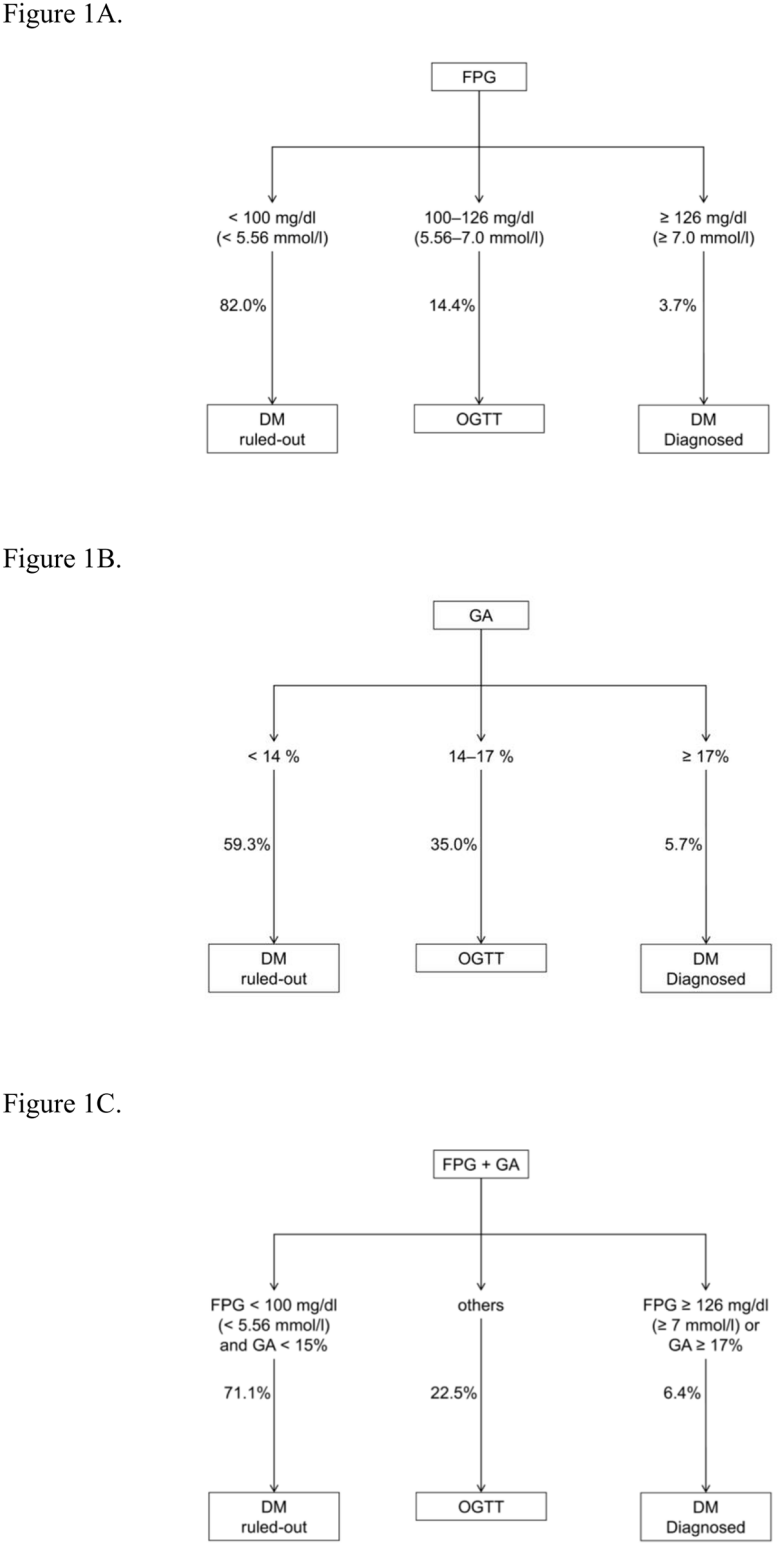
Screening strategies to find diabetes by OGTT. Proportions of population in specific diagnostic category were shown. (A) By impaired fasting glucose (IFG) criteria, that is, fasting plasma glucose (FPG) < 100 mg/dL (5.56 mmol/L) to exclude and FPG ≥ 126 mg/dL (7.0 mmol/L) to diagnose diabetes. The sensitivity, specificity, positive predictive value (PPV), negative predictive value (NPV), false positive rate (FPR), and false negative rate (FNR) for this strategy were 78.8%, 100%, 100%, 98.1%, 0%, and 21.2%, respectively. (B) By serum glycated albumin (GA) at 14% and 17%, the sensitivity, specificity, PPV, NPV, FPR, and FNR for this strategy were 83.3%, 98.2%, 80.9%, 98.5%, 1.8%, and 16.7%, respectively. (C) By combine IFG and GA criteria, that is, FPG < 100 mg/dL (5.56 mmol/L) and GA < 15% to exclude diabetes and FPG ≥ 126 mg/dL (7.0 mmol/L) or GA ≥ 17% to diagnose diabetes. The sensitivity, specificity, PPV, NPV, FPR, and FNR for this strategy were 85.6%, 98.2%, 81.3%, 98.7%, 1.8%, and 14.4%, respectively.

The use of two GA cutoffs with and without FPG cutoffs for diagnosis of diabetes was examined. Table 3A–3C and Fig 1A–1C describe the performance of serum GA alone and in combination with IFG criteria as well as the performance of IFG criteria alone for diagnosis of diabetes by the OGTT. Using the IFG criteria of FPG < 5.56 mmol/L to exclude and FPG ≥ 7 mmol/L to diagnose diabetes (Fig 1A), the sensitivity, specificity, and OGTT% were 78.8%, 100%, and 14.4%, respectively. As shown in Table 3A, the choice of cutoff GA value was associated with a tradeoff between sensitivity/specificity and OGTT%. For example, the sensitivity and OGTT% increased as the cutoff to exclude diabetes decreased. However, the specificity and OGTT% increased as the cutoff to diagnose diabetes increased. Based on the performance of the IFG alone criteria, cutoff values which can minimize OGTT% with sensitivity greater than 80% and specificity greater than 95% were selected. The proposed algorithms are summarized in Fig 1. As shown in Fig 1B, use serum GA < 14% to exclude and ≥ 17% to diagnose diabetes, the sensitivity, specificity and OGTT% were 83.3%, 98.2% and 35%, respectively. As seen in Fig 1C, use of FPG < 5.56mmol/L and serum GA < 15% to exclude and of FPG ≥ 7.0 mmol/L or serum GA ≥ 17% to diagnose diabetes, the sensitivity, specificity and OGTT% were 85.6%, 98.2% and 22.5%, respectively.

**Table 3:** Performance of (A) different cutoffs of serum glycated albumin (GA) alone, (B) impaired fasting glucose (IFG) only, and (C) IFG plus different cutoffs of serum GA, ie. fasting plasma glucose (FPG) < 100 mg/dl and serum GA < cutoffs to exclude diabetes mellitus (DM), and FPG ≥ 126 mg/dl or serum GA ≥ cutoffs to diagnose DM.

GA cutoffs to exclude DM	GA cutoffs to diagnose DM	OGTT%	Sensitivity (%)	Specificity (%)	PPV (%)	NPV (%)	FPR (%)	FNR (%)
(A) Serum GA alone								
13%	16%	59.3	94.7	94.8	62.8	99.5	5.2	5.3
	17%	63.6	94.7	98.2	82.8	99.5	1.8	5.3
	18%	65.6	94.7	99.2	91.9	99.5	0.8	5.3
**14%**	16%	30.7	83.3	94.8	59.8	98.4	5.2	16.7
	**17%**	**35.0**	**83.3**	**98.2**	**80.9**	**98.5**	**1.8**	**16.7**
	18%	36.9	83.3	99.2	90.9	98.5	0.8	16.7
15%	16%	9.8	74.2	94.8	57.0	97.5	5.2	25.8
	17%	14.1	74.2	98.2	79.0	97.6	1.8	25.8
	18%	16.0	74.2	99.2	89.9	97.7	0.8	25.8
(B) Impaired fasting glucose only								
FPG < 100 mg/dl	FPG ≥ 126 mg/dl	14.4	78.8	100.0	100.0	98.1	0.0	21.2
(C) Impaired fasting glucose plus GA cutoffs								
14%	16%	35.6	89.4	94.8	61.5	99.0	5.2	10.6
	17%	39.7	89.4	98.2	81.9	99.0	1.8	10.6
	18%	41.2	89.4	99.2	91.5	99.0	0.8	10.6
**15%**	16%	18.3	85.6	94.8	60.4	98.6	5.2	14.4
	**17%**	**22.5**	**85.6**	**98.2**	**81.3**	**98.7**	**1.8**	**14.4**
	18%	23.9	85.6	99.2	91.1	98.7	0.8	14.4
16%	17%	10.9	81.8	94.8	59.3	98.3	5.2	18.2
	18%	15.0	81.8	98.2	80.6	98.3	1.8	18.2

%OGTT, percentage require an OGTT; DM, diabetes mellitus; FNR, false negative rates; FPG, fasting plasma glucose; FPR, false positive rates; GA, glycated albumin; IFG, impaired fasting glucose; NPV, negative predictive value; PPV, positive predictive value.

Using serum GA alone as the criterion for diagnosis of diabetes (serum GA < 14% to exclude and ≥ 17% to diagnose diabetes), 22 subjects with diabetes and 26 subjects without diabetes were falsely diagnosed. The clinical features of the false negative and the false positive subgroups were therefore compared; those with diabetes but who were falsely diagnosed had higher BMI, WC, and serum albumin concentrations (Table 4).

**Table 4:** Clinical characteristics of subgroups with false negative or false positive results using GA alone criteria for diagnosis of diabetes, ie. serum GA < 14% to exclude and ≥ 17% to diagnose diabetes.

	False Negative	False Positive	*p*
*n*	22	26	
Age (years)	60.4 ± 8.1	60.9 ± 11.6	0.495
Sex (male/female)	6/16	8/18	0.791
Family history of diabetes (N, %)	4 (18.2%)	6 (23.1%)	0.677
BMI (kg/m^2^)	26.8 ± 3.2	22.6 ± 2.1	<0.001
WC (cm)	88.3 ± 8.3	78.5 ± 6.9	<0.001
Serum albumin (g/dL)	4.3 ± 0.4	4.1 ± 0.4	0.036
Serum creatinine (mg/dL)	1.0 ± 0.2	1.1 ± 0.2	0.213

Means ± SDs were shown for continuous variables. BMI, body mass index; GA, glycated albumin; WC, waist circumference.

## Discussion

This report presents several new findings. First, findings of this study reveal that GA can be measured accurately in plasma or serum samples, and in samples from non-fasting subjects. Second, quadratic relationships were demonstrated among serum GA, plasma glucose, and HbA1c for subjects with and without diabetes. Third, this study is the first to develop two screening strategies to diagnose diabetes according to serum GA values. Both strategies were found capable of reducing the need for an OGTT and, when compared with the performance of IFG criteria alone, performed well. These findings support the use of serum GA as an alternative to HbA1c under conditions wherein use of the latter is known to be doubtful for diagnosis of diabetes.

The two strategies developed in the present study for diagnosis of diabetes according to serum GA values are illustrated in Fig 1. Use of serum GA alone to screen for diabetes (Fig 1B) is convenient for patients because fasting is not required. When compared with the IFG criteria alone (Fig 1A), use of serum GA is associated with better sensitivity (83.3% *vs*. 78.8%) and a lower false-negative percentage (16.7 *vs*. 21.2); however, more subjects will require an OGTT (35% *vs*. 14.4%). On the other hand, if fasting is not a concern, use of FPG with serum GA as a screening strategy improves use of IFG criteria alone as a screening strategy (Fig 1C). In particular, sensitivity is increased from 78.8% to 85.6% and the false negative percentage reduced from 21.2 to 14.4 with an acceptable increase in the percentage of subjects requiring an OGTT (IFG criteria alone, 14.4; FPG plus serum GA criteria, 22.5). Two other studies using serum GA to diagnose diabetes have been reported but only one GA cutoff value was used in these studies. In the first which examined Japanese subjects [34], the sensitivity and specificity for GA as the diagnostic criterion were 83.3% and 83.3%, respectively. In the second which examined Chinese subjects [35], the sensitivity and specificity for GA as the diagnostic criterion were 73.3% and 80.1%, respectively. The present study employed two cutoffs, one to exclude and one to diagnose diabetes, such that sensitivity and specificity were improved significantly (sensitivity 83.3% and specificity 98.2%). It should be noted that the authors of the study of Chinese subjects [35] suggested that measurements of both FPG and serum GA (FPG at 6.1 mmol/L and GA at 17.1% as the cutoffs) had better identified those who had diabetes; however, those subjects were high-risk group for diabetes (i.e., those with FPG ≥ 6.1 mmol/L, with random plasma glucose ≥ 7.8 mmol/L, or with a positive family history of diabetes). By contrast, the present study was performed with subjects derived from the overall community.

The good performance of serum GA in the diagnosis of diabetes by the OGTT in the present study is supported by the findings of the two studies described above [34, 35], with the area under the ROC curve ranging from 0.86 to 0.91. In the present study, the optimal cutoff for serum GA was 15%, with a sensitivity of 74% and a specificity of 85%. Similarly, the optimal cutoff for plasma GA in the study of Japanese subjects was 15.5%, with a sensitivity of 83.3% and a specificity of 83.3% [34]. In the study of Chinese subjects, a GA cutoff point of 15.7% was observed, with a sensitivity of 73.3% and a specificity of 80.3% [35]. Taken together, these findings favor an optimal serum GA cutoff for 15–16% for Asian populations.

Findings of the present study support the convenience to patients of GA measurements. As with HbA1c measurement, fasting is not required, as was evidenced by the finding that serum GA values were similar in fasting and post-prandial samples. The correlation coefficient for serum GA and plasma GA was very high (0.9987), indicating that GA can be measured in either specimen type. By contrast, HbA1c can only be measured in whole blood samples.

In a previous study conducted by the authors of this report [5], strategies using HbA1c to diagnose diabetes were proposed. Using HbA1c alone (HbA1c < 5.9% [41 mmol/mol] to exclude and ≥ 7.0% [53 mmol/mol] to diagnose diabetes), the performance was found to be better than using serum GA alone (HbA1c criteria *vs*. serum GA criteria: sensitivity of 89.1% *vs*. 83%, false negative percentage of 10.9 *vs*. 16.7, and OGTT% of 26.5 *vs*. 35). However, using IFG plus HbA1c criteria (FPG < 5.56 mmol/L and HbA1c < 6.1% [43 mmol/mol] to exclude and FPG ≥ 5.56 mmol/L and HbA1c ≥ 7% [53 mmol/mol] to diagnose diabetes), the performance was similar to IFG plus GA criteria (IFG plus HbA1c criteria *vs*. IFG plus GA criteria: sensitivity of 85.2% *vs*. 85.6%, false negative percentage of 10.9 *vs*. 14.4, and OGTT% of 18.9 *vs*. 22). These findings strongly support the measurement of serum GA as a reasonable alternative to the measurement of HbA1c in the diagnosis of diabetes.

Serum GA values are influenced by several factors associated with albumin turnover independently of glycemia status, such as liver cirrhosis and thyroid dysfunction. For example, thyroid hormone is known to promote albumin catabolism. Furthermore, serum GA correlates positively and significantly with serum TSH and negatively and significantly with free T3and free T4 [36]. Additionally, GA values are reported to be influenced by age and nutritional status independently of glycemic status in non-diabetic subjects with end-stage renal disease [37]. In diabetic patients with CKD, nephrotic-range proteinuria decreases GA values regardless of the glycemic status whereas non-nephrotic range proteinuria has no significant influence on GA values [38].

As indicated above, serum GA reflects short-term (2–3 weeks) mean glycemic values whereas HbA1c reflects longer-term (2–3 months) mean glycemic values [18, 19]. *In vivo* non-enzymatic glycation of albumin is approximately nine times that of hemoglobin, with the glycation reaction occurring ten times more rapidly for albumin [39]. Because glycation of plasma proteins occurs more readily than glycation of hemoglobin, glycated albumin should serve well in the evaluation of glycemic control for both type 1 and type 2 diabetes [40]. Because serum GA is not affected by erythrocyte survival time [20], GA measurements appear appropriate for patients with anemia or hemoglobinopathies [18]. Accordingly, serum GA has been suggested as an alternative marker to HbA1c in subjects with nephropathy [41], retinopathy [42], gestational diabetes, and iron-deficiency anemia [43]. Although HbA1c measurements underestimate glycemic values in diabetic patients on dialysis, GA measurements accurately reflect glycemic status for these patients [21–23, 44, 45].

In the present study, serum GA was found to be positively associated with age. A similar association was observed in other studies [34, 37, 46]. The finding that serum GA is inversely correlated with both BMI and WC was surprising but is also in agreement with the findings of other studies [47–50]. Since GA is also correlated with high-sensitivity C-reactive protein, this observation may be explained by the chronic, low-grade inflammation observed in obese subjects and which is known to increase the rate of albumin catabolism and to reduce rate of albumin synthesis [51, 52]. The negative association between serum GA, BMI, and WC may also explain why subjects with diabetes who were falsely diagnosed by using serum GA alone had higher BMI and WC. The quadratic relationships of serum GA to FPG, to 2-h PG, and to HbA1c observed in the present study are in agreement with the findings of a study of subjects receiving health examinations [34]. Subjects with diabetes were diagnosed with the disease for the first time during that study and during the present study. The observed curvilinear relationships are therefore proposed to be attributable to the capacity of serum GA to measure recently-occurring (within the previous 2–3 weeks) hyperglycemia. By contrast, in studies for which subjects with a relatively stable glycemic status were recruited, linear relationships between serum GA and HbA1c were observed [24, 34, 35, 45, 46]. For example, the DCCT study [24] recruited subjects with type 1 diabetes well-controlled by medical treatment and who had relatively stable glycemic values as compared with the subjects in the present study with recently-diagnosed diabetes.

Strengths of the present study include its large sample size, permitting feasible and reliable statistical analyses to be performed. The present study is also comprehensive. For example, the associations between GA and several potential confounding factors were analyzed, the conversion of serum GA to HbA1c values was possible, and different diagnostic strategies for use of serum GA in the diagnosis of diabetes by the OGTT were developed, including one with a single GA cutoff value, one with two cutoff GA values, and one using both IFG and GA criteria. However, the findings of this study, which included Han Chinese adults only, may not prove applicable to other ethnic groups. Nonetheless, OGTT is not repeated in the present study when the results are in the diabetic range, which is different from the recommendations by the ADA and is another limitation of the present study.

## Conclusions

In the diagnosis of diabetes mellitus, serum GA measurements with or without IFG criteria are more sensitive than IFG criteria alone when assessing the need for an OGTT. The optimal cutoff value for serum GA alone in diagnosis of diabetes is 15%. Use of FPG < 100 mg/dL (5.56 mmol/L) with serum GA < 15% to exclude and FPG ≥ 126 mg/dL (7.0 mmol/L) or serum GA ≥ 17% to diagnose diabetes further increases the sensitivity of the screening strategy and reduces the proportion of subjects requiring an OGTT. Measurement of serum GA is a more practical and time-saving than performance of an OGTT and can be used to determine the need for an OGTT in the diagnosis of diabetes mellitus. Serum GA presents a useful alternative to HbA1c under conditions wherein the latter does not reflect glycemic status accurately in the diagnosis of diabetes.

## Supporting Information

S1 Fig The relationship between serum glycated albumin (GA) and (A) age, (B) gender, (C) serum albumin, (D) body mass index, and (E) waist circumference in subjects without diabetes. The correlation coefficient between serum GA and age was 0.27 (p < 0.001). The regression coefficient between serum GA and age per 10 years was 0.31 (p < 0.001). Means ± SDs of serum GA were 14.0 ± 2.6% for women and 13.9 ± 2.6% for men (p = 0.26). The correlation coefficient between serum GA and albumin was -0.1179 (age-adjusted p = 0.001). The age-adjusted regression coefficient between serum GA and serum albumin was -0.32, suggesting that every 1 g/dl increase in serum albumin is associated with a decrease in serum GA of 0.32%. The correlation coefficient between serum GA and BMI was -0.2391 (age-adjusted p < 0.001). The age-adjusted regression coefficient between serum GA and BMI was -0.12, indicating that every 1 kg/m^2^ increase in BMI is associated with a decrease in serum GA of 0.12%. The correlation coefficient between serum GA and WC was -0.1607 in subjects without diabetes (age-adjusted p < 0.001). The age-adjusted regression coefficient between serum GA and WC was -0.04, suggesting that every 1 cm increase in WC is associated with a decrease in serum GA of 0.04%.
(PDF)

S2 Fig The method of sample collection on the value of glycated albumin (GA). (A) The relationship of GA in matched serum and plasma samples (r = 0.9987, p < 0.001). Serum GA = 0.98 x plasma GA + 0.29. (B) Serum GA in fasting blood samples and in blood samples collected 2 hours after OGTT. The means ± SDs of GA were 17.9 ± 6.7% in fasting samples and 18.0 ± 6.5% in samples collected 2 hours after OGTT (p = 0.8). (C) Plasma GA in fasting blood samples and post-prandial blood samples collected 2 hours after a meal. The means ± SDs of GA were 15.8 ± 4.3% in fasting samples and 15.8 ± 4.3% in post-prandial blood samples (p = 0.8).
(PDF)

S3 Fig The relationship between serum glycated albumin, (A) fasting plasma glucose, (B) plasma glucose 2 hours after OGTT, and (C) hemoglobin A1c. Open circles, subjects without diabetes; close circles, subjects with diabetes. Diabetes was diagnosed by an oral glucose tolerance test.
(PDF)
