# Supplementary material for: Serum Glycated Albumin to Guide the Diagnosis of Diabetes Mellitus
Source: PLoS One. 2016 Jan 14;11(1):e0146780. doi: 10.1371/journal.pone.0146780 (PMC4713060; doi:10.1371/journal.pone.0146780)

Supplementary Figure 1A.

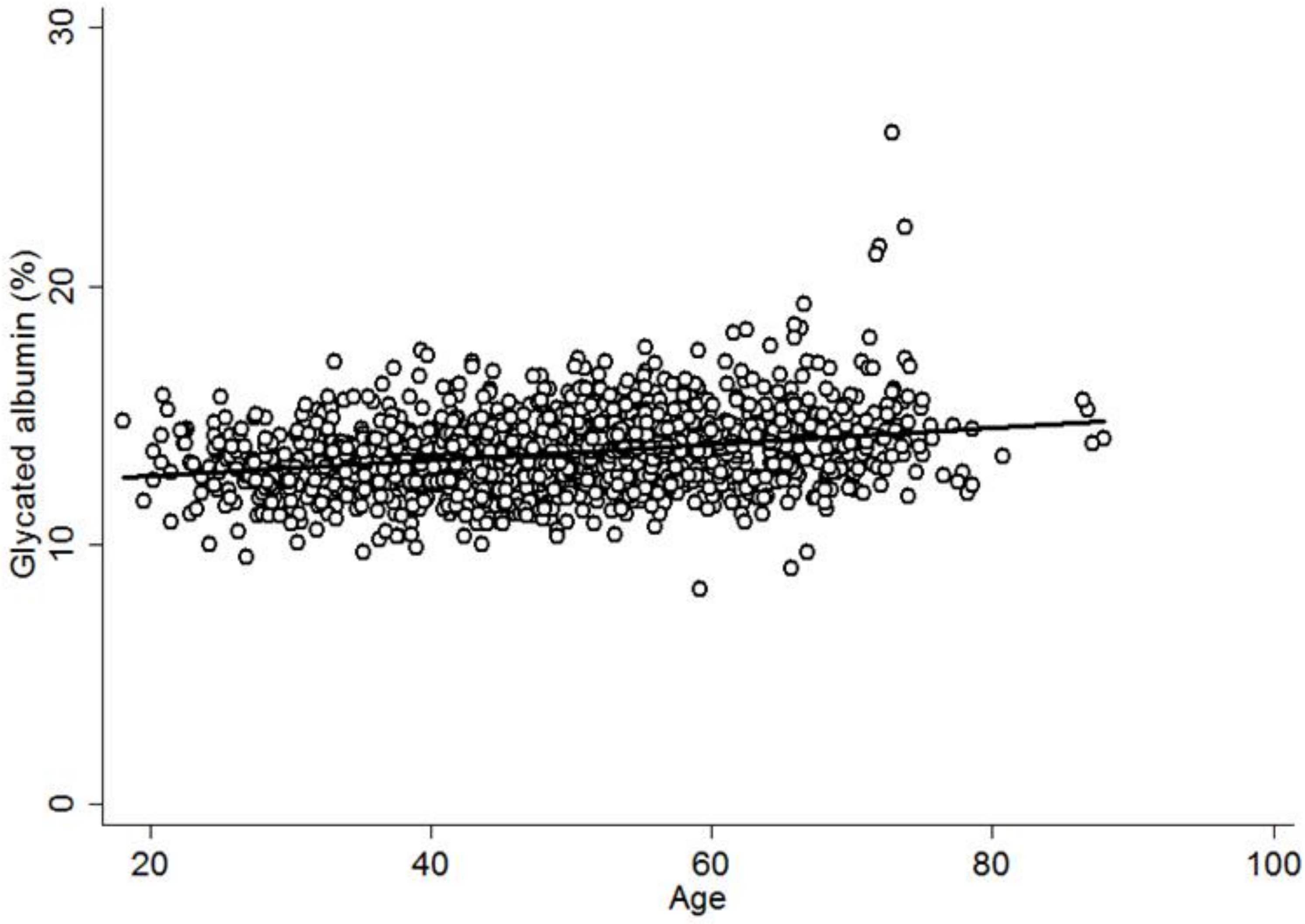

Supplementary Figure 1B.

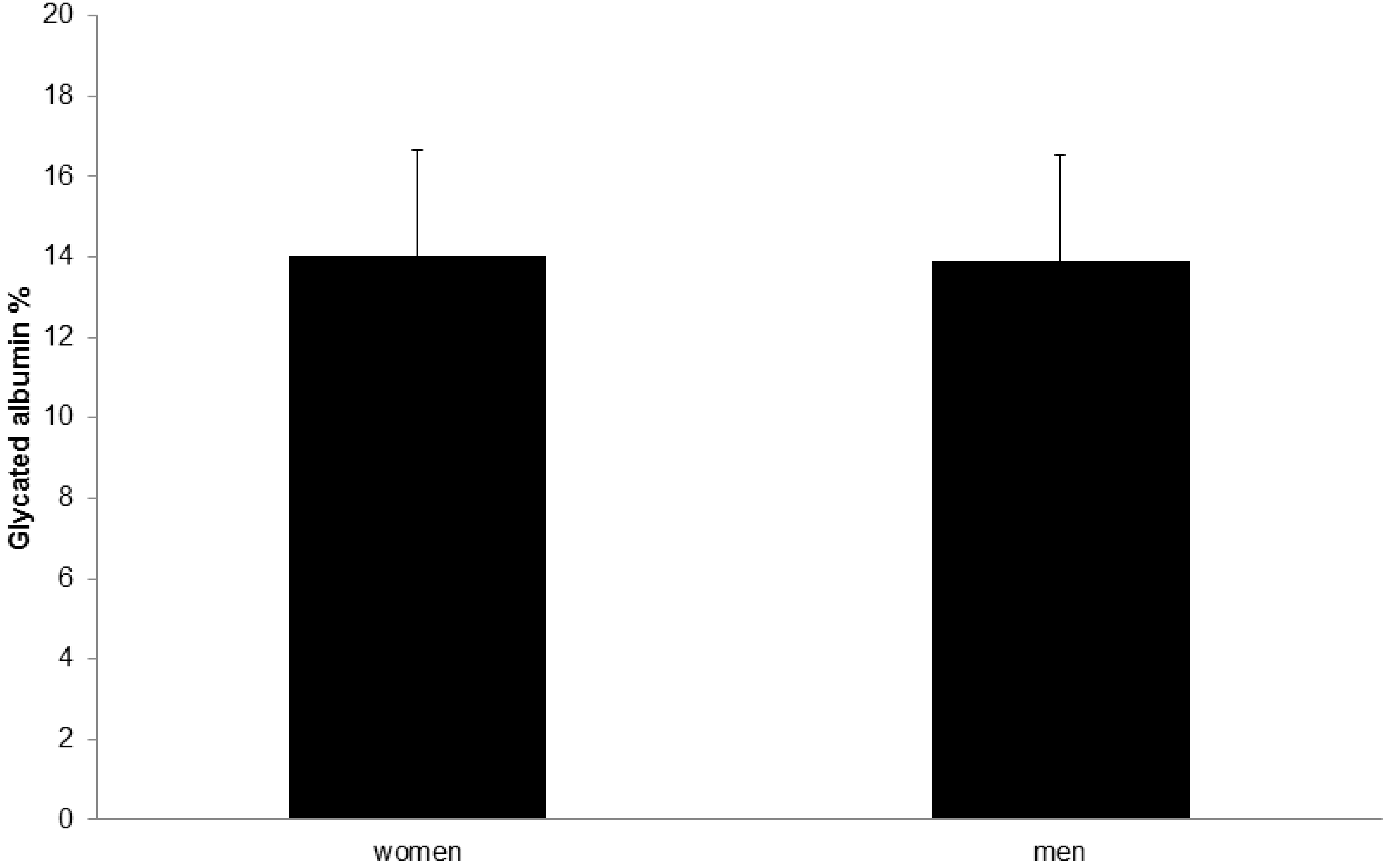

Supplementary Figure 1C.

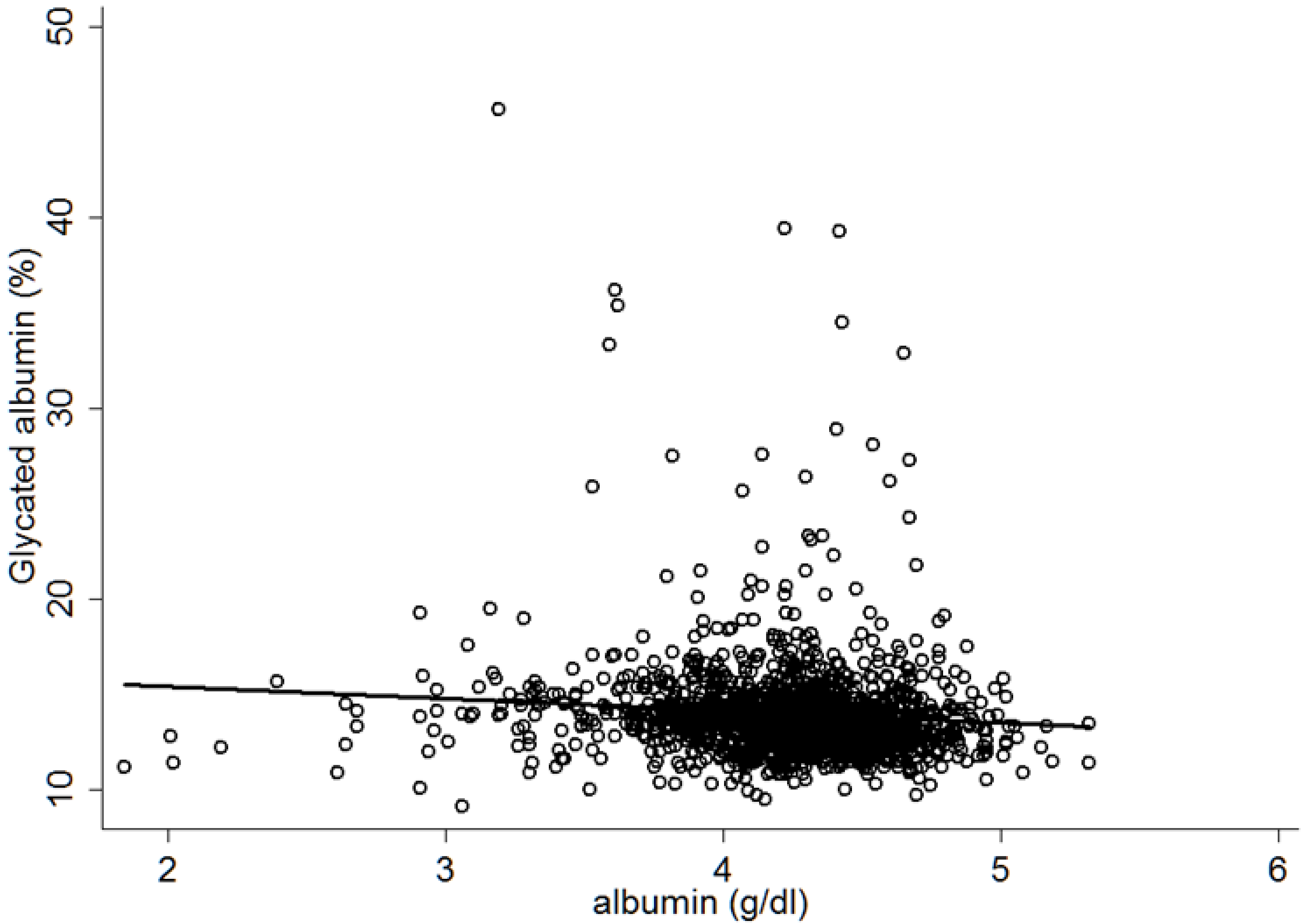

Supplementary Figure 1D.

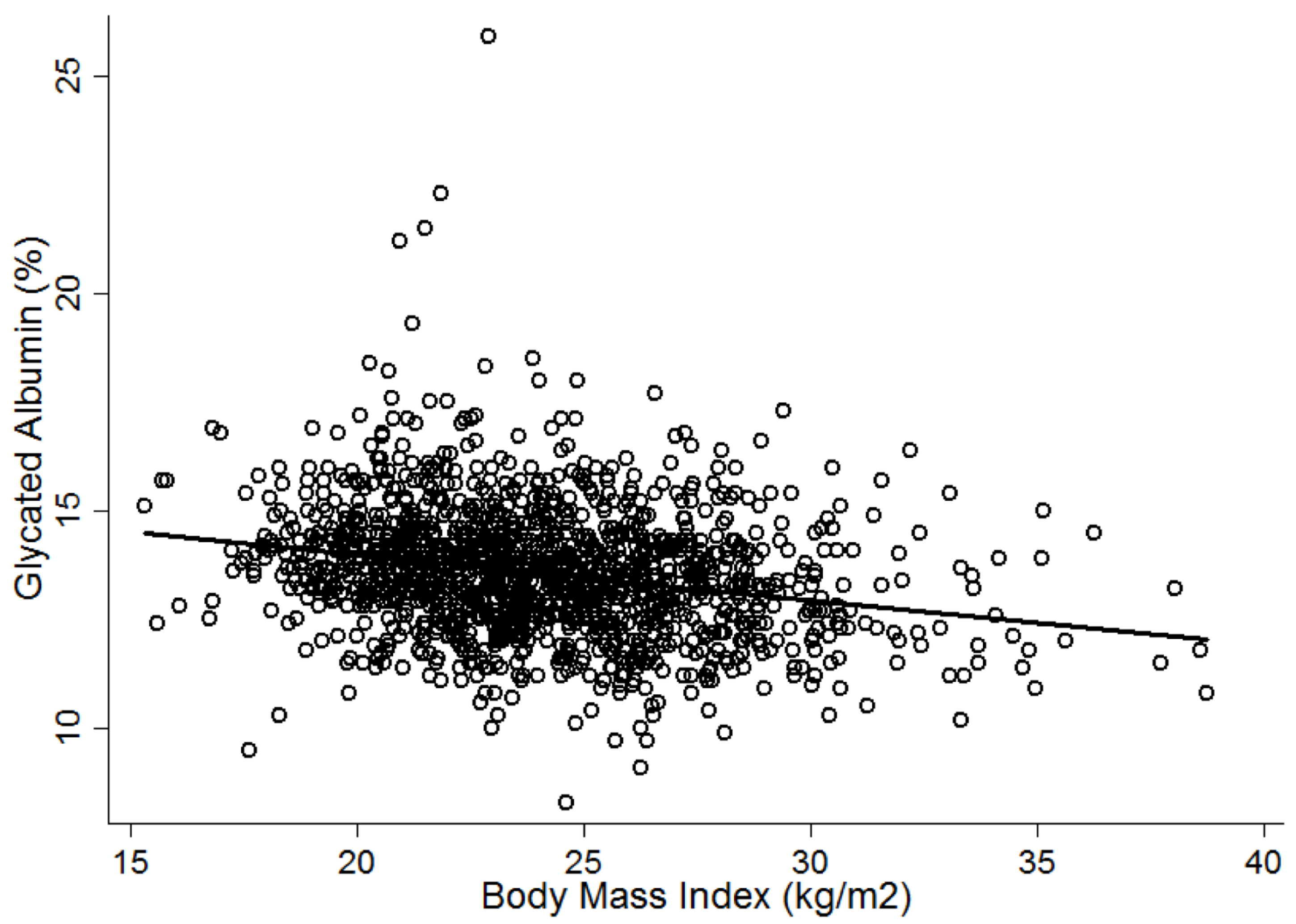

Supplementary Figure 1E.

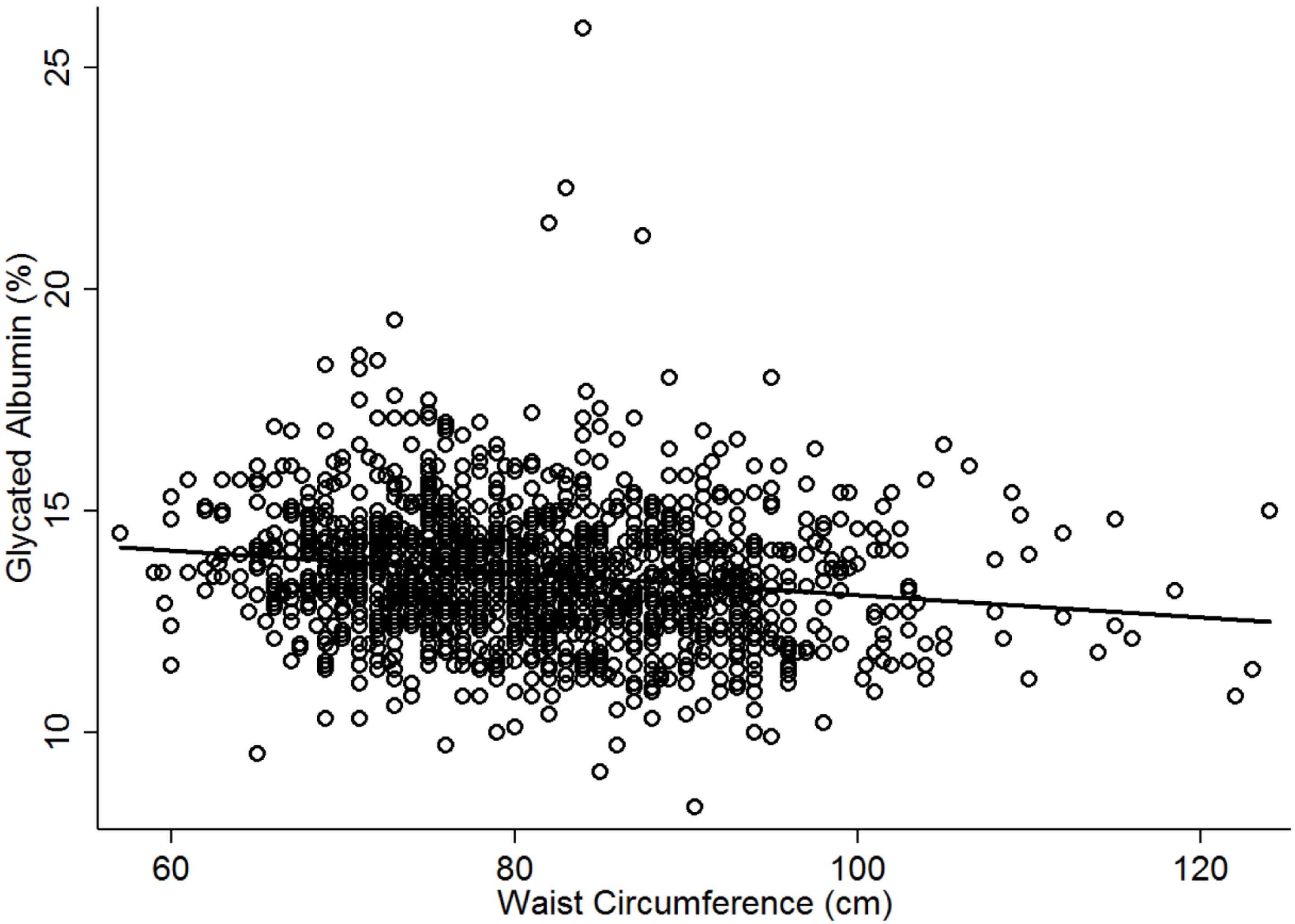

Supplement: S1 Fig — The correlation coefficient between serum GA and age was 0.27 (p < 0.001). The regression coefficient between serum GA and age per 10 years was 0.31 (p < 0.001). Means ± SDs of serum GA were 14.0 ± 2.6% for women and 13.9 ± 2.6% for men (p = 0.26). The correlation coefficient between serum GA and albumin was -0.1179 (age-adjusted p = 0.001). The age-adjusted regression coefficient between serum GA and serum albumin was -0.32, suggesting that every 1 g/dl increase in serum albumin is associated with a decrease in serum GA of 0.32%. The correlation coefficient between serum GA and BMI was -0.2391 (age-adjusted p < 0.001). The age-adjusted regression coefficient between serum GA and BMI was -0.12, indicating that every 1 kg/m2 increase in BMI is associated with a decrease in serum GA of 0.12%. The correlation coefficient between serum GA and WC was -0.1607 in subjects without diabetes (age-adjusted p < 0.001). The age-adjusted regression coefficient between serum GA and WC was -0.04, suggesting that every 1 cm increase in WC is associated with a decrease in serum GA of 0.04%. (PDF) [file pone.0146780.s001.pdf]
