# Supplementary figures and images for: Serum Glycated Albumin to Guide the Diagnosis of Diabetes Mellitus
Source: PLoS One. 2016 Jan 14;11(1):e0146780. doi: 10.1371/journal.pone.0146780 (PMC4713060; doi:10.1371/journal.pone.0146780)

**Supplementary Figure 2A.**

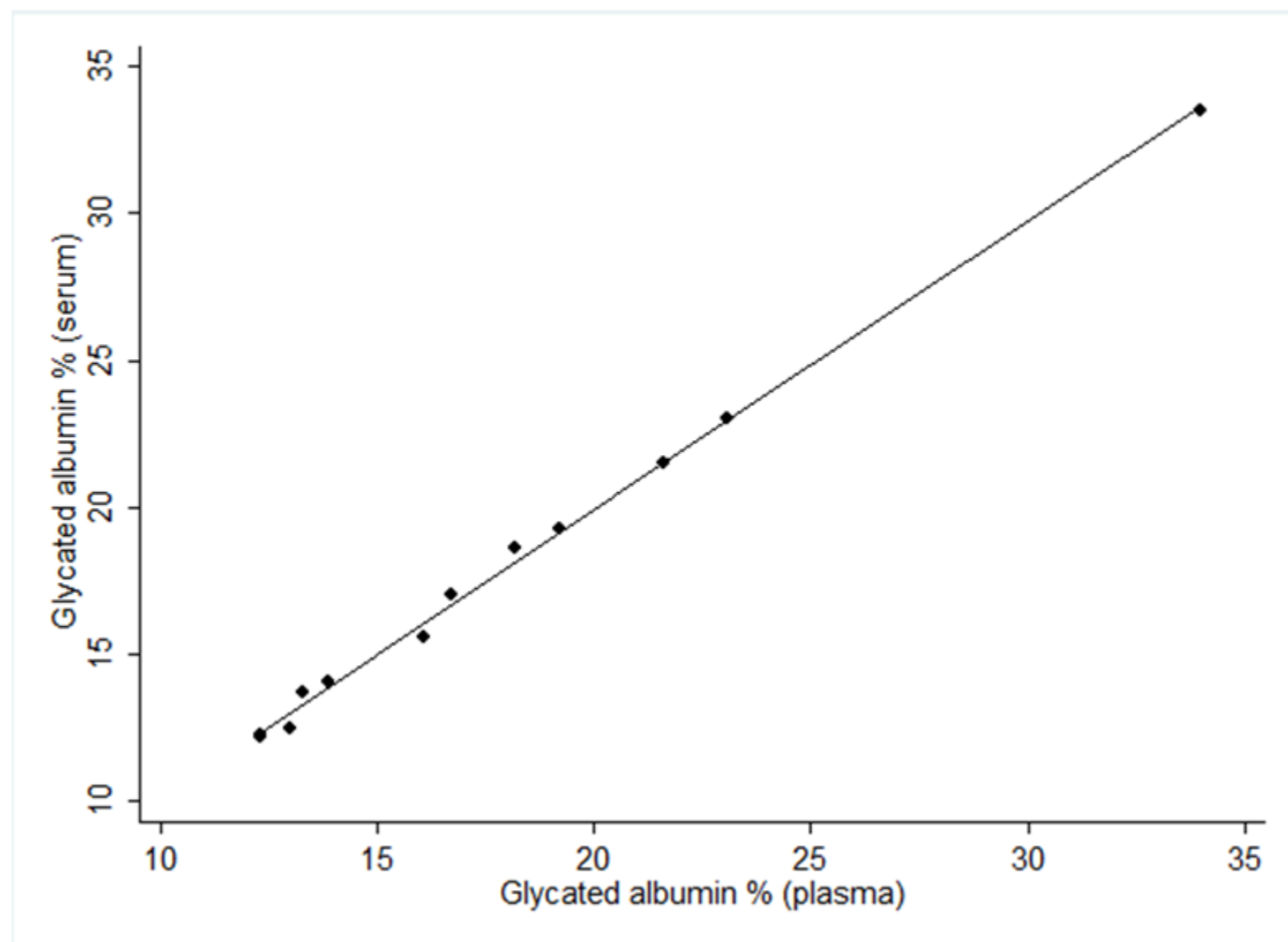

## Supplementary Figure 2B.

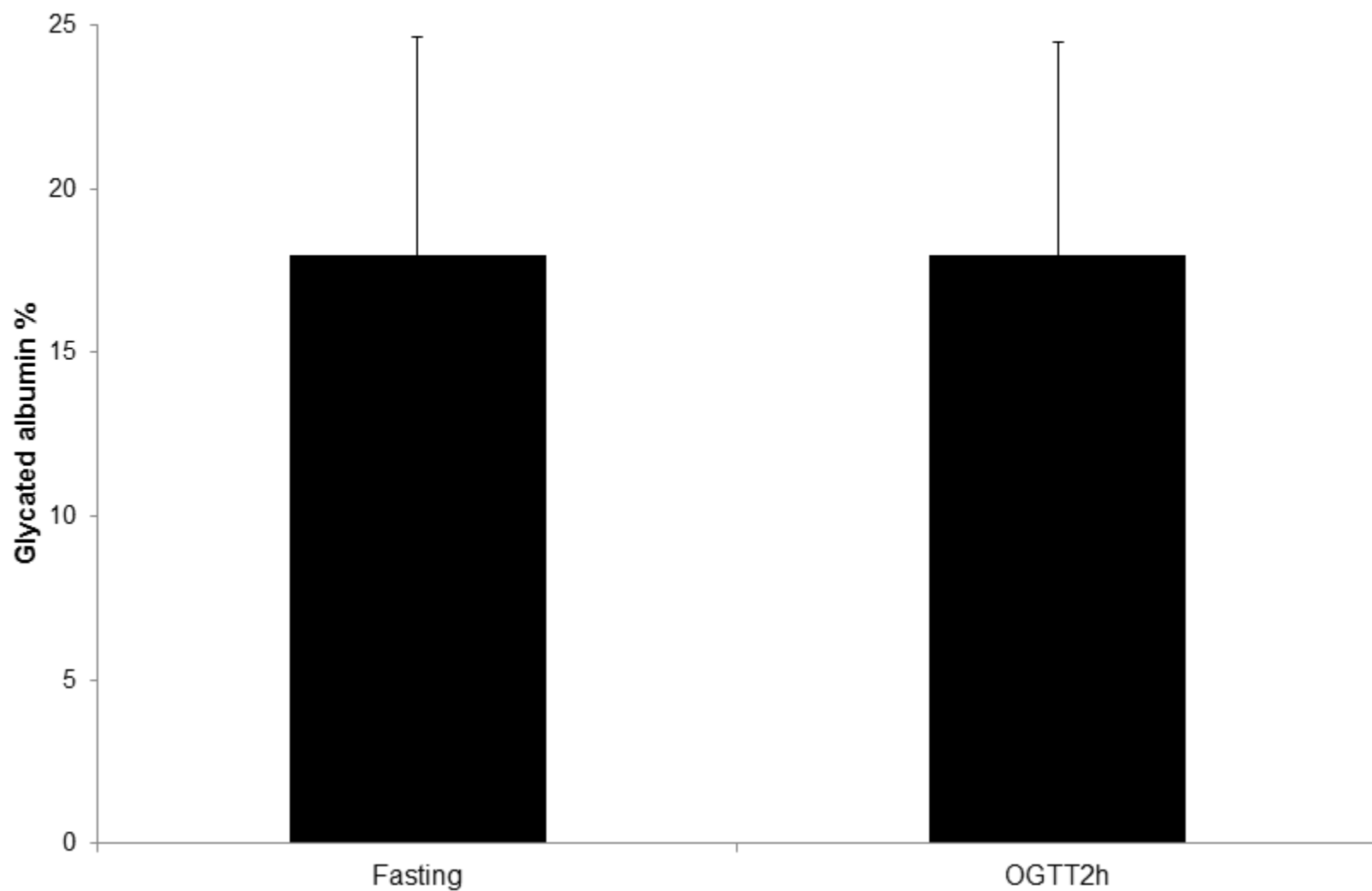

**Supplementary Figure 2C.**

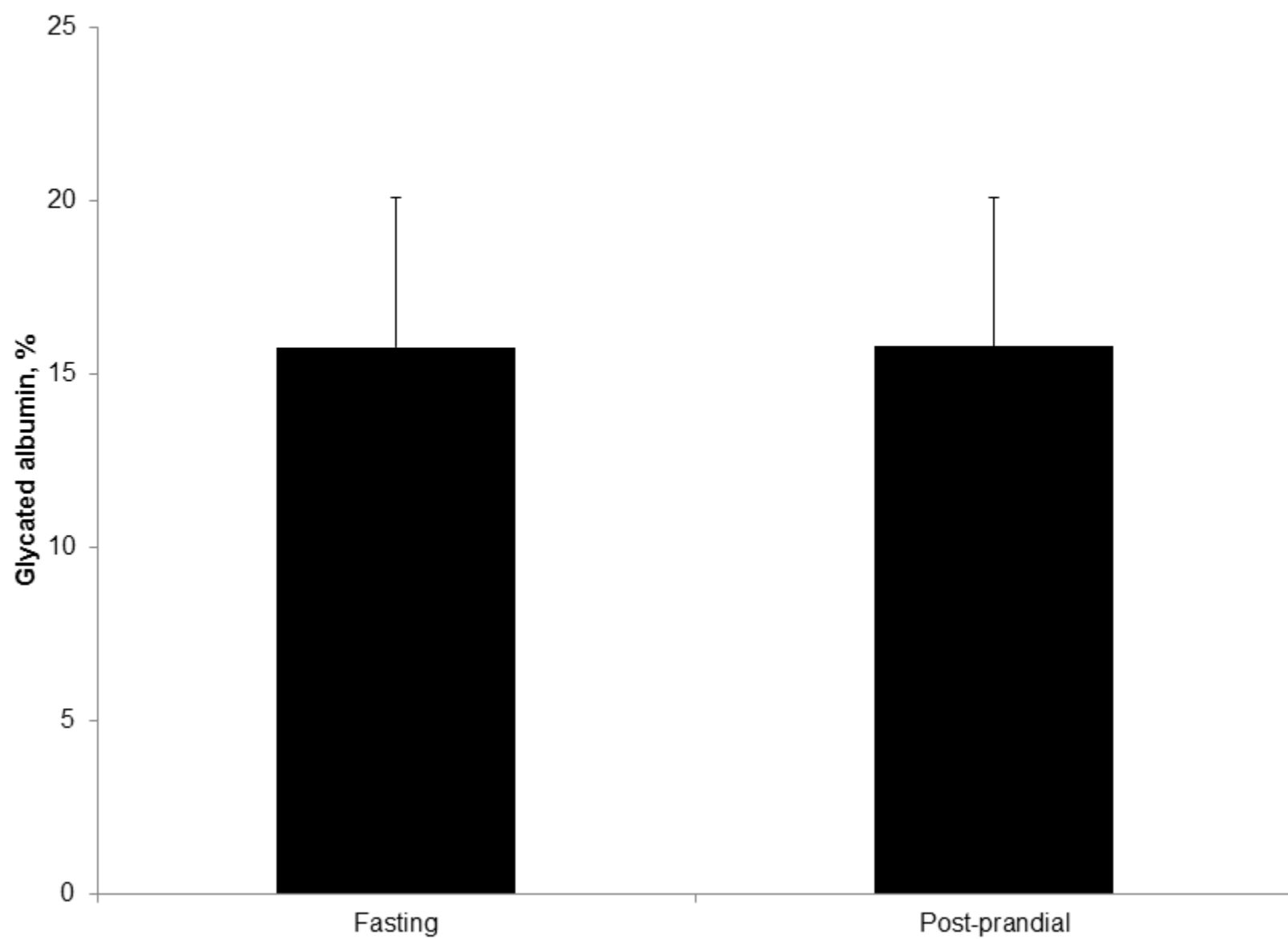

Supplement: S2 Fig — (A) The relationship of GA in matched serum and plasma samples (r = 0.9987, p < 0.001). Serum GA = 0.98 x plasma GA + 0.29. (B) Serum GA in fasting blood samples and in blood samples collected 2 hours after OGTT. The means ± SDs of GA were 17.9 ± 6.7% in fasting samples and 18.0 ± 6.5% in samples collected 2 hours after OGTT (p = 0.8). (C) Plasma GA in fasting blood samples and post-prandial blood samples collected 2 hours after a meal. The means ± SDs of GA were 15.8 ± 4.3% in fasting samples and 15.8 ± 4.3% in post-prandial blood samples (p = 0.8). (PDF) [file pone.0146780.s002.pdf]

**Supplementary Figure 3A.**

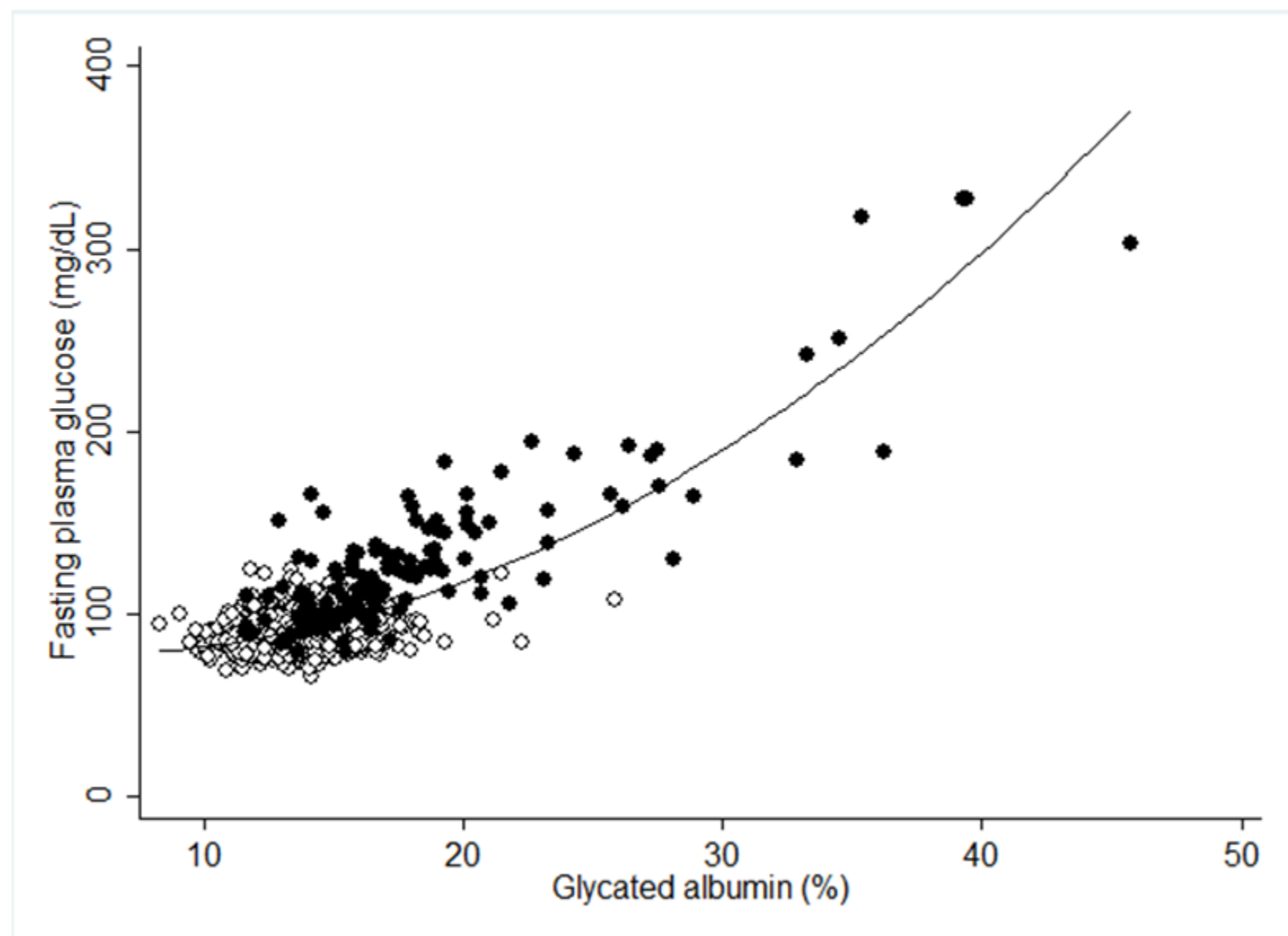

**Supplementary Figure 3B.**

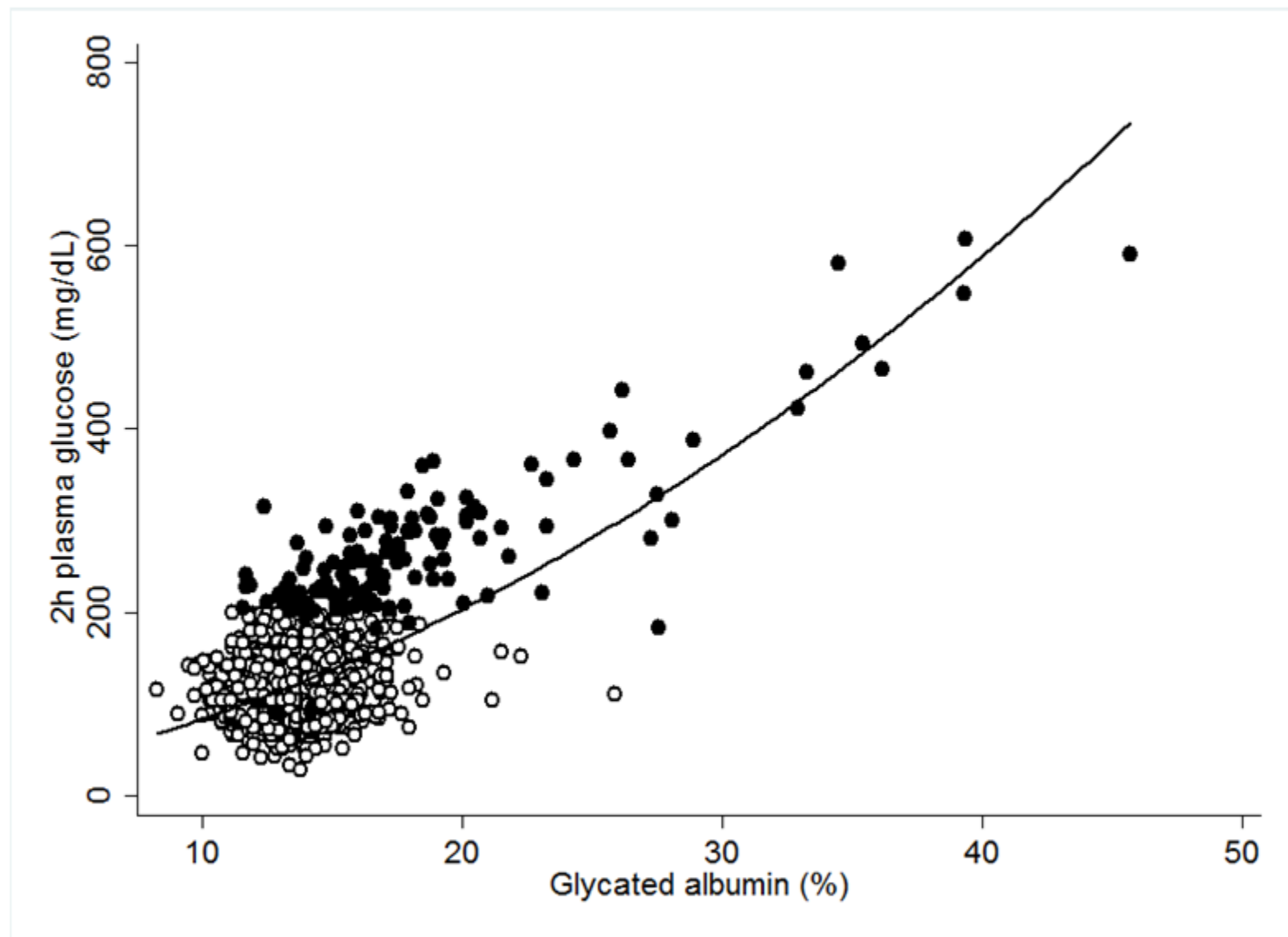

**Supplementary Figure 3C.**

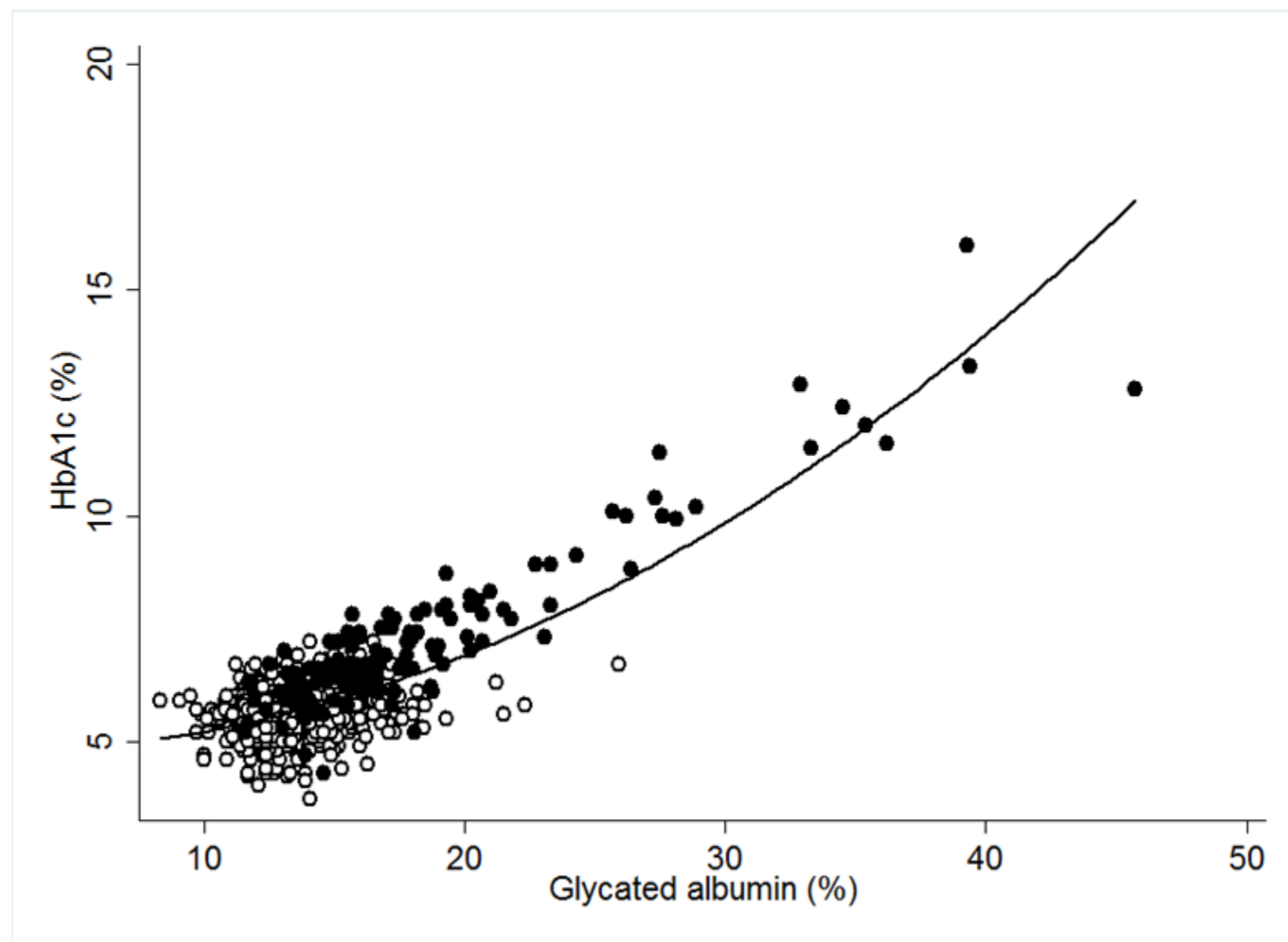

Supplement: S3 Fig — Open circles, subjects without diabetes; close circles, subjects with diabetes. Diabetes was diagnosed by an oral glucose tolerance test. (PDF) [file pone.0146780.s003.pdf]
